# The protean presentations of XK disease (McLeod syndrome): a case series with new observations and updates on previously reported families

**DOI:** 10.3389/fnins.2024.1408105

**Published:** 2024-09-09

**Authors:** Ruth H. Walker, Mariana Barreto, James R. Bateman, M. Leonor Bustamante, Graham Chiu, Scott Feitell, Beat M. Frey, Patricio Guerra, Sofia Guerrero, Hans H. Jung, Fernando Maldonado, Eduardo Meyer, Marcelo Miranda, Emelie McFarland, Patricia Oates, Gorka Ochoa, Karin Olsson, Martin Paucar, Jonatan Alvarez Proschle, Esther M. Sammler, Monica Troncoso, Rachel Wu-Wallace, Leo Young, Sunitha Vege, Connie M. Westhoff, Adrian Danek

**Affiliations:** ^1^Department of Neurology, James J. Peters Veterans Affairs Medical Center, New York, NY, United States; ^2^Department of Neurology, Mount Sinai School of Medicine, New York, NY, United States; ^3^Diagnosis Foundation, Santiago, Chile; ^4^Mental Health Service Line and the Mental Illness Research, Education, and Clinical Center, W.G. (Bill) Hefner Salisbury Veterans Affairs Medical Center, Salisbury, NC, United States; ^5^Department of Neurology, Wake Forest University School of Medicine, Winston-Salem, NC, United States; ^6^Human Genetics Program, Faculty of Medicine, Biomedical Sciences Institute, University of Chile, Santiago, Chile; ^7^Rheumatologist, Palmerston North, New Zealand; ^8^Rochester Regional Health, Sands-Constellation Heart Institute, Rochester, NY, United States; ^9^Regional Blood Transfusion Service, Swiss Red Cross, Zurich, Switzerland; ^10^School of Medicine, University San Sebastián, Puerto Montt, Chile; ^11^Hospital De Castro, Los Lagos, Chile; ^12^Department of Neurology, University Hospital, Zurich, Switzerland; ^13^Servicio Salud Reloncaví, Puerto Montt, Chile; ^14^Clínica Meds, Lo Barnechea, Chile; ^15^New York Blood Center Enterprises, New York, NY, United States; ^16^Department of Neurology, Karolinska University Hospital, Stockholm, Sweden; ^17^Department of Clinical Neuroscience, Karolinska Institutet, Stockholm, Sweden; ^18^Independent Researcher, Puerto Varas, Chile; ^19^University of Dundee, School of Medicine, Dundee, Scotland; ^20^Hospital San Borja Arriaran, University of Chile, Santiago, Chile; ^21^UCLA Semel Institute for Neuroscience & Human Behavior, Los Angeles, CA, United States; ^22^Department of Neurology, LMU University Hospital, LMU Munich, Munich, Germany

**Keywords:** McLeod, XK, chorea, acanthocytosis, neurodegeneration

## Abstract

XK disease is a very rare, multi-system disease, which can present with a wide spectrum of symptoms. This disorder can also be identified pre-symptomatically with the incidental detection of serological abnormalities when typing erythrocytes in peripheral blood, or on other routine laboratory testing. Increasing awareness of this disorder and improved access to genetic testing are resulting in increasing identification of affected patients and families. Here we provide updates to some previously-reported families and patients and provide additional clinical details. We also report four new cases with a variety of presentations, one of whom had a novel mutation.

## Introduction

XK disease, formerly known as McLeod syndrome, is a primarily neurodegenerative disorder which additionally affects cardiac and skeletal muscle, peripheral nervous system, liver, and spleen (Jung et al., 1993). Inheritance is X-linked, typically affecting middle-aged men, although occasionally carrier females develop symptoms. Most neurological manifestations are movement disorders, typically chorea, tics, but also parkinsonism and dystonia, however seizures and neuropsychiatric symptoms can be presenting features. Psychiatric symptoms can include psychosis, delusions, depression, obsessive-compulsive features, and others. Subjects are increasingly being identified by incidental genomic screening in addition to the detection of elevated transaminases and creatine kinase (CK), red blood cell (RBC) acanthocytosis, or by weak expression of Kell blood group system antigens with absence of Kx antigen, i.e. the McLeod phenotype, when typing of red blood cells by blood transfusion centers (Lee et al., 2000; Redman et al., 1999). If subjects are transfused with Kell+ blood they may develop anti-Kell antibodies which can result in massive hemolysis of subsequent Kell+ transfusions, thus transfusion with appropriately matched blood type is critical. The acanthocytosis does not appear to result in major hematologic consequences apart from a mild hemolytic anemia. The diagnosis can be made either by RBC phenotyping or by identification of a pathogenic variant of XK. If the diagnosis is made initially by genetic methodology, RBC phenotyping should also be performed for confirmation (Jung et al., 1993).

We present details of two families; one large kinship from Chile related to two affected brothers who have been previously reported (Gantenbein et al., 2011; Miranda et al., 2012; Miranda et al., 2007), and the family and neurological history of a patient previously published in brief (Floch et al., 2021; Boppana et al., 2023; Lim et al., 2022). We also present clinical details of a patient whose blood was examined as part of blood analyses from a series of patients with VPS13A disease (chorea-acanthocytosis) and XK disease (Siegl et al., 2013). We report the first case of XK disease diagnosed in a person of African ancestry, who had a novel mutation. This patient suffered from severe behavioral issues which were primarily eating-related, and we discuss the strategies employed to manage these issues. We also describe two unrelated patients with an identical mutation and widely differing presentations, emphasizing the apparent absence of genotype-phenotype correlations; one of these presented with a sleep disorder, recognized to be a clinically significant feature of XK disease (Lim et al., 2022; Danek et al., 2001; Munter et al., 2023), and the other with a seizure disorder and severe depression.

## Methods

We obtained the latest clinical information regarding previously published subjects, in addition to those recently identified. All subjects gave permission for publication of their clinical data.

### Case series

#### Family 1

**FIGURE 1:**
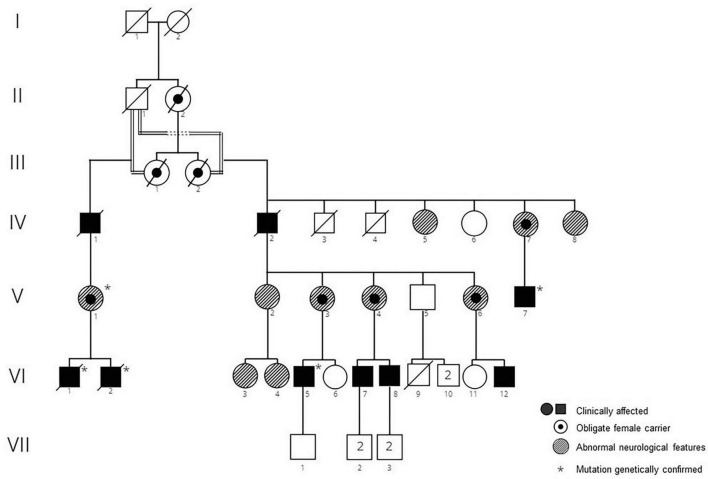
Pedigree of family 1. Clinically affected men, obligate carrier women, and women with atypical neurologic featurs are shown.

In this large family with significant consanguinity, a pathogenic variant in the *XK* gene, c.856_860del (p.Leu286Tyrfs*16), (previously termed 938-942delCTCTA) was originally identified in two members of, this pedigree (Figure 1). These individuals have been previously reported in detail; VI-1 [(Miranda et al., 2012) case 2 (Miranda et al., 2007); patient C (Gantenbein et al., 2011)] and VI-2 [case 1 (Miranda et al., 2007)]. The family had emigrated in 1857 from Höchstberg in current Baden-Württemberg, Germany, as part of a wave of German emigration to Chile.

IV-1:There is no clinical information about the patient but it is assumed that he was affected, being an obligate carrier.

IV-2: This 76-year-old man had a 10-year history of chorea and a 2-year history of seizures. Cognitively he was clinically normal. Peripheral blood smear demonstrated acanthocytosis.

IV-5: This woman was reported to suffer from muscular atrophy. Further details are not available.

IV-7: This woman who was an obligate mutation carrier was reported to suffer from progressive blindness. Further details are not available.

IV-8: At 87 years old this woman was found to have pigmentary retinopathy (RP) and dementia.

V-1, V-3, V,6; These women were obligate mutation carriers and were reported to have abnormal neurological features, however details are not available.

V-2: This woman was observed to have a peripheral neuropathy. Her CK was normal; and she had 1-2 acanthocytes/field on peripheral blood smear.

V-4: This woman was an obligate mutation carrier. At 39 years old she was neurologically asymptomatic and had a normal neurological examination except for decreased vibratory sensation in lower extremities. Electrophysiology revealed a moderate, axonal-predominant sensory-motor polyneuropathy. CK was normal, however she had acanthocytosis on peripheral blood smear.

V-7: This man presented with chorea and cognitive decline at age 49. Total CK was 1964 IU/L (normal < 250 IU/L). Brain MRI identified mild diffuse atrophy, predominantly supratentorial, and microangiopathic in appearance. Echocardiogram was normal. On examination he had mild chorea of the lower extremities, oral dyskinesia, and mild dysarthria.

VI-1 and VI-2: The clinical details of these brothers are published in detail elsewhere [VI-1 (Miranda et al., 2012), case 2 (Miranda et al., 2007), patient C (Gantenbein et al., 2011); VI-2, case 1 (Miranda et al., 2007)]. Briefly, VI-1 developed chorea at the age of 47 which later progressed to parkinsonism, in addition to mild cognitive issues, obsessive-compulsive features, and emotional lability. IV-2 presented with paranoid psychosis at age 23 and chorea 6 years later, albeit following the use of anti-psychotic medications.

VI -3: This woman was reported to suffer from *anorexia nervosa.* No further details are available.

VI-4: This 29-year-old woman was neurologically asymptomatic, and normal on neurological examination apart from decreased vibratory sensation in lower extremities. Magnetic resonance imaging (MRI) was reported to show moderate cerebellar atrophy. Her CK was normal.

VI-5: This man first noticed abnormal movements when he was 15 years old. At age 36 he continues to work at a high cognitive level without difficulties. He reported some mild obsessive-compulsive behaviour such as checking and counting. On examination he was cognitively normal with a Montreal Cognitive Assessment (MoCA) score of 28. There was mild chorea, motor tics, and his gait was impaired with frequent stumbling and tripping. Deep tendon reflexes were absent throughout. No feeding dystonia was observed and there was no history of seizures. CK was markedly elevated (5000 IU/L; (normal < 250 IU/L); only 6% acanthocytes were found in the peripheral blood smear after osmotic stress. Brain MRI showed very mild caudate atrophy.

VI-7: This man was neurologically asymptomatic at the age of 20, with a normal neurological examination except for decreased vibratory sensation in lower extremities. Electrophysiology demonstrated moderate, axonal-predominant sensory-motor polyneuropathy. CK was markedly elevated at 4,524 IU/L (normal < 250 IU/L), and acanthocytosis was reported in peripheral blood.

**FIGURE 2:**
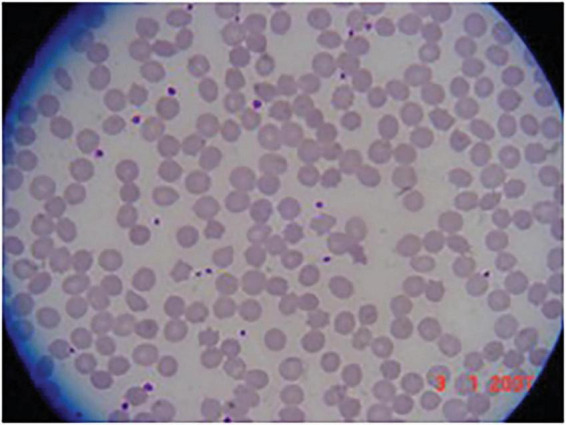
Acanthocytosis in patient VI-8. Peripheral blood smear showing acanthocytes.

VI-8: At 13 years old this boy was evaluated for the incidental laboratory finding of elevated transaminases. Intelligence and psychomotor milestones in infancy were normal. His main symptom was fatigue. Muscle strength and deep tendon reflexes were normal; the only neurological abnormality was a decrease in vibration sense in both legs. Electrophysiology revealed a moderate, axonal-predominant sensory-motor polyneuropathy. CK was 1,213 IU/L (normal < 250 IU/L); there was RBC acanthocytosis (Figure 2). He continued to be neurologically asymptomatic at the age of 17, although there was persistent CK elevation (11,827 IU/L). He was subsequently lost to medical follow-up.

VI-12: This man is severely affected and is bedridden 15 years after symptom onset. Further details are not available.

#### Family 2

**FIGURE 3:**
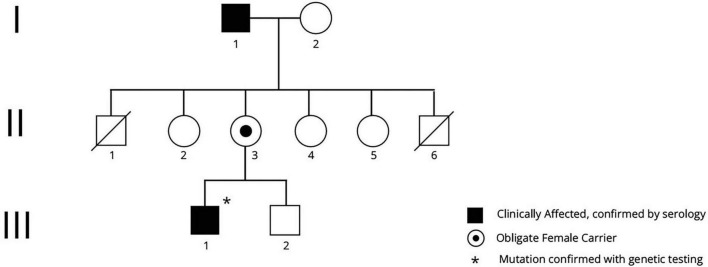
Pedigree of family 2. Clinically affected men, in whom the diagnosis was serologically confirmed, are shown, with the obligate carrier woman. Male subjects II-1 and II-6 both died of causes likely related to XK disease.

This family (Figure 3) was of northern European ancestry. Several other family members were reported to have cardiac disease and were potentially also affected, based upon serological testing of RBCs, however further clinical details are not available. II-1 died in his sleep aged 54. II-6 was reported to have died at age 20 from a cardiac cause. No affected females are reported.

I-1: In his early 50s this man developed muscle weakness, tics, severe depression, and was found to have the McLeod RBC phenotype. He died at the age of 60 due to cardiac disease, possibly related to ventricular tachycardia.

III-1: This 44-year-old man presented with severe congestive heart failure (CHF) with an ejection fraction of 10% and runs of non-sustained supraventricular tachycardia. He had been found at the age of 6 to have the McLeod RBC phenotype, following the diagnosis of his maternal grandfather (I-1). Neurological examination at age 44 demonstrated intact strength and reflexes, with no involuntary movements. His CHF was satisfactorily managed with an implantable cardioverter defibrillator (AICD) (Boppana et al., 2023). A sleep study demonstrated severe periodic limb movements of sleep (PLMS) and sleep apnea (case 6 (Lim et al., 2022)). CK was normal (225 IU/L; normal 32–267 IU/L); aspartate aminotransaminase (AST) was normal (34 IU/L; normal 0–35 IU/L), while alanine aminotransferase (ALT) was mildly elevated (71 IU/L, normal 0–35 IU/L). Brain MRI was unremarkable.

Sequencing of *XK* identified a pathogenic mutation c.577A > T encoding for a premature stop codon (p.Lys193Ter) (case 2 (Floch et al., 2021)).

#### Case A

This 57-year-old white male (presumptively of Northern European ancestry) was first noted to have 33% acanthocytosis on peripheral blood film. One year later, he was presented with acute but transient dysphagia, and was noted to have continuous choreoathetoid movements affecting all four limbs, trunk, and head, in addition to wasting of intrinsic muscles of the hands and feet. Cognitive functioning was preserved, however, he displayed symptoms of schizophreniform psychosis, most notably the belief that he had a clairvoyant ability to communicate with his deceased brother. (It is unknown as to whether his brother was also affected.) In addition to the acanthocytosis, a raised CK was the only abnormal blood result.

Nerve conduction testing confirmed presence of a severe sensory motor axonal peripheral polyneuropathy with only mild neurogenic changes, and no myopathic features. Sural and superficial peroneal sensory responses were bilaterally absent, as were ulnar sensory responses.

Brain MRI showed mild generalized atrophy and minor signal abnormalities in the caudate nucleus, and an incidental pituitary macroadenoma. 24-h-electrocardiogram and echocardiogram were normal.

Over the next 3 years, prior to his death, there were major issues with mood, labile affect, and delusions, the latter of which he denied. His myopathy progressed with severe distal wasting of all four limbs, he became wheelchair-dependent, and developed parkinsonism in combination with choreathetoid movements. His cognitive functioning deteriorated, and he developed increased emotional lability and suicidal ideation.

Targeted genetic testing following identification of the McLeod RBC phenotype identified a point mutation in exon 3, c.1023G > A which encodes for a premature stop codon. This mutation was previously reported in an unrelated patient (Supple et al., 2001).

This subject’ blood was analyzed as part of a study of erythrocyte membrane properties in patients with acanthocytosis due to various neurogenetic disorders (including VPS13A disease and pantothenate kinase-associated neurodegeneration [PKAN]) (Siegl et al., 2013). As his was the single sample with XK disease, no statistical comparison with the other disorders or controls was possible.

#### Case B

A 59-year-old Black American man had a progressive history of involuntary movements and behavioral issues. He reported difficulties with verbal disinhibition and tics beginning in his 40’s, which had a significant impact on his interpersonal relationships. The family was African-American with no family history of neurological issues.

Neurological examination at age 54 showed generalized chorea, with motor impersistence. Deep tendon reflexes were trace in all extremities and plantar response was flexor bilaterally. All other neurological aspects were normal. Speech was soft and he tended to repeat himself. Gait was unsteady and staggering.

He was reported to violently act out his dreams, which was attributed to his diagnosis of PTSD following military service. He was very paranoid and there were many instances of severe behavioral disturbances resulting in multiple legal issues with trespassing and larceny. He had compulsive vomiting and was admitted to a long-term care facility as he was unable to live at home due to hoarding, defecating, and urinating in the home with no interest or acknowledgment of the need to clean, bathe, or eat properly.

At age 58, he was seen for psychotherapy to address multiple psychiatric symptoms and complaints. Presenting problems included symptoms related to trauma and depression including nightmares, negative alterations with beliefs and emotional state, irritability, concentration, sleep disturbance, shame, anhedonia, sadness, psychomotor agitation, and feelings of worthlessness. He also endorsed obsessive and perseverative thoughts about food. Behaviorally, he exhibited unwanted eating and feeding symptoms including food hoarding, binge eating, vomiting, and reactions of anger when food was taken away from him. Eating symptoms were complicated by his reported difficulties with extreme polydipsia, hyperphagia, and dysphagia. Despite placement of a feeding tube and nighttime feedings, he continued to have vomiting and malnutrition. He primarily described feelings of distress and shame surrounding vomiting and vocal tics, which negatively impacted his ability to socialize with others.

Psychotherapy treatment focused on increasing self-esteem by separating his self-identity from his medical symptoms and use of coping strategies, specifically positive imagery, to decrease vocal tics which were exacerbated by stress. He reported benefits from these interventions. Due to sanitary concerns from food hoarding as well as behavior towards staff regarding food, he was placed on a behavioral modification plan that specified mealtimes, snack limits, rewards for good behavior, as well as rules for room cleaning, treatment of staff, and other floor mates (e.g., not stealing food from other long-stay individuals). Staff reported a decrease in behavioral difficulties when enforcing the behavior plan.

Peripheral blood smear was reported to show 15–20% acanthocytes; CK was elevated at 1629 IU/L, as was AST. Brain MRI showed caudate atrophy. He had a reported history of atypical chest pain but echocardiography was normal.

He carried a diagnosis of “neuroacanthocytosis” for many years until genetic sequencing was performed and identified a novel mutation in *XK*, c.484C > T encoding for a premature stop codon (p.Gln162Ter). RBC antigen typing showed weak expression of Kell system antigens and Kx- phenotype (Table 1), indicating a McLeod phenotype.

**TABLE 1:** Summary of mutations, their serological effects, and relevant previous publications related to each case.

	Mutation	Effect of mutation	Erythrocyte phenotype	ISBT erythrocyte phenotype	Previous publications
Family 1	c.856_860del	p.Leu286Tyrfs*16	Kx 0, K1 0, K2 +, K3 0, K4 0	XK-1; KEL:-1,2,-3,-4	Gantenbein et al., 2011; Miranda et al., 2007; Miranda et al., 2012
Family 2	c.577A > T	p.Lys193Ter	K–k+w Kp(a–b+w) Kx-	XK-1; KEL:-1,2w,-3,4w	Boppana et al., 2023; Floch et al., 2021; Lim et al., 2022
Case A	c.1023G > A	p.Trp341Ter	Kx absent (no other information available)	XK:-1	Siegl et al., 2013
Case B	c.484C > T	p.Gln162Ter	K k+^w^, Kp(a b+^w^), Js(a b+^w^) Kx–	XK:-1; KEL:-1,2w,-3,4w,-6,7w	N/A
Case C	c.397C > T	p.Arg133Ter	K+/k-^wk^, Kp(a-b+^wk^) and Kx-; very weak expression of k and Kp^b^	XK:-1; KEL:1,2w,-3,4w	N/A
Case D	c.397C > T	p.Arg133Ter	Absent K, Kpa, and Kpb; weak expression of k	KEL:-1,2,3w,4w	N/A

We include the revised RBC nomenclature as per the International Society of Blood Transfusion (IBST).

#### Case C

A 42-year-old man of Scottish and English ancestry was referred to a rheumatologist for evaluation of elevated CK which had been present for at least 12 years. There was no family history of neurological disorders. He had reported having “restless legs syndrome” for 10 years, with recent worsening of involuntary movements during the day. He did not report any issues with thinking or memory. His main complaint was of very restless and active sleep. On examination constant fasciculations were noted of his calf muscles in addition to subtle involuntary foot movements; strength and sensation were intact, deep tendon reflexes were reduced.

Brain MRI was uninterpretable due to his involuntary movements. 30% acanthocytosis was noted on peripheral blood smear. He had persistently raised troponin T of 28–62 ng/L (normal - 0–13 ng/L) and cardiac MRI showed global impairment of systolic function (left ventricular ejection fraction 55%), with a calcium coronary computed tomography (CT) score of 0. A sleep study was performed following reports of severe abnormal movements during sleep, and demonstrated REM sleep behavior disorder (RBD) and obstructive sleep apnea (body mass index [BMI] 28.3). Continuous positive airway pressure (CPAP) was not tolerated and worsened his involuntary movements.

Targeted genetic testing following identification of the McLeod RBC phenotype showed c.397C > T in exon 2 of *XK* encoding for a premature stop codon (p.Arg133Ter) (Table 1). This mutation was not found in his mother, thus this appears to be a *de novo* mutation. His 3 brothers had normal Kell and XK antigen expression.

#### Case D

This 52-year-old Finnish man with no family history of neurological disease, presented with generalized seizures at age 38. He suffered from recurrent severe depressive episodes and suicidality and was referred for weakness, myalgia, progressive balance difficulties and involuntary movements. The time course for the onset of the involuntary movements was not clearly defined, however these worsened around age 46. Upon examination he had no deficits on comprehensive cognitive evaluation. Examination of eye movements revealed broken smooth pursuit with horizontal end-gaze nystagmus, mild slowness of saccades in both vertical and horizontal directions and hypometric saccades. There was mild chorea of the extremities and trunk, humming, and moderate dysarthria. He had distal muscle atrophy and mild, generalized weakness, absent deep tendon reflexes, and a normal sensory examination; there was no feeding dystonia, dysphagia or weight loss.

**FIGURE 4:**
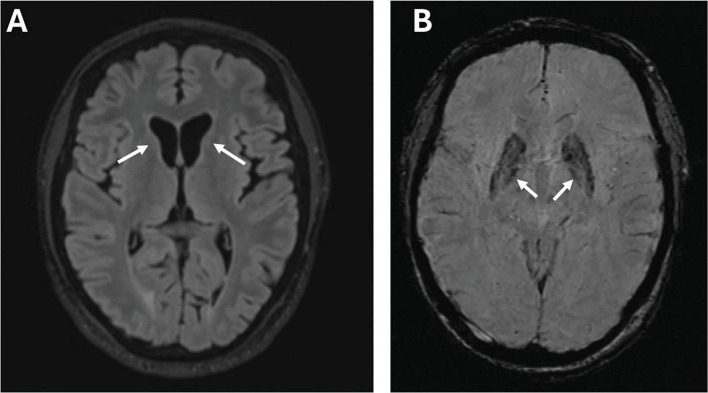
Imaging features of Case D. Brain MRI of case D at age 53 years, showing marked atrophy of the caudate nuclei bilaterally in a T1-weighted scan (arrows) **(A)**; susceptibility-weighted images (SWI) sequence showing abnormally increased iron deposition in the globus pallidus (arrows) **(B)**.

Laboratory tests revealed elevated CK, myoglobin, and lactate dehydrogenase levels but no signs of hemolysis. Brain MRI showed caudate atrophy, cortical atrophy (Figure 4), and mildly increased iron accumulation in globus pallidus. Cardiac evaluation was unremarkable. On electrophysiological study he had a sensorimotor axonal neuropathy, and biopsy from the right tibialis anterior muscle a mild myopathy.

His seizures were refractory to treatment with carbamazepine and subsequently lamotrigine, but were ultimately controlled on a combination of valproic acid, perampanel and cenobamate. His depression has also been refractory to treatment with an antidepressant.

On peripheral blood smear only 2% of erythrocytes were acanthocytes. On targeted genetic testing he was found to have c.397C > T, p.R133X in exon 2 of *XK* (Blueprints Genetics, Finland). This variant was absent in his mother and brother, thus was a *de novo* mutation. Kell antigens were absent or weakly expressed, and Kx was absent (Table 1), consistent with the McLeod phenotype.

## Discussion

XK disease is an X-linked recessive disorder which can resemble other progressive neurodegenerative disorders, specifically Huntington’s disease (HD). The diagnosis can be suspected when patients with chorea or other movement disorders are also noted to have seizures, myopathy, or peripheral neuropathy, with laboratory findings or elevated CK, transaminases, or acanthocytes. There continues to be no evidence of a genotype-phenotype correlation. Even within families, such as Family 1, there is a spectrum of symptoms, which can be cognitive, psychiatric, or neurological.

We highlight here the multi-faceted aspects of XK disease: Presentations include dysphagia (case A), severe psychiatric and behavioral issues (cases B, D), seizures (case D), and disordered sleep (case C).

Case B is remarkable for the severity of his psychiatric and behavioral issues. Psychiatric issues in XK disease can vary widely; in many patients these are minimal, however occasional patients with severe issues are reported (Jung and Haker, 2004), similar to those in this and case D. Management can be very challenging, however cognitive-behavioral therapy (CBT) appears to have been relatively beneficial for this patient.

Case C was being evaluated for chronic CK elevation, and his main neurological presentation was with a sleep disorder. Both this patient and case B were noted to have RBD early in their disease course.

A spectrum of sleep disorders, including PLMS, RBD, and sleep apnea, has been identified as a significant cause of morbidity and mortality in XK disease (Danek et al., 2001; Lim et al., 2022; Munter et al., 2023). Prospective laboratory sleep studies may be informative in people diagnosed with XK disease especially given the contributions of sleep disorders to cardiac and cognitive impairment.

Case D presented with seizures, and subsequently with severe psychiatric issues, with less prominent chorea and other features. His seizures were initially resistant to treatment but were ultimately controlled with complex polypharmacy. His presentation and disease course have been strikingly different from that of case C, with whom he shares a genetic variant. This variant, p.Arg133Ter (R133X), so far the most commonly-occurring variant, and has been reported previously in a number of unrelated patients from Europe (Danek et al., 2001; Torres et al., 2022; Dotti et al., 2000; Klempíř et al., 2008; Bansal et al., 2008; Díaz-Manera et al., 2018) (n = 8) and one Japanese patient (Murakami et al., 2019; Urata et al., 2019).

Of note, the specifics of the McLeod RBC phenotype can be variable with the same genetic variant, although this can be challenging to compare due to variations in testing antibodies used. The phenotypic variability of red blood cells as assessed by conventional serology is mainly due to technical issues (subjective read-out of hemagglutination assays, gel versus tube technique, variable avidity of polyclonal antibodies). This might be avoided by flow cytometry or fluorescence-activated cell sorting (FACS) assessment of red blood cells, because the applied monoclonal antibodies are more standardized. In addition FACS analysis recognizes double red cell population of Kx+/Kx- red cells in female carriers (Miranda et al., 2007; Jung et al., 2001).

Most patients reported have been of northern European ancestry, with a small number being East Asian or Hispanic. Case B is the first patient reported with African ancestry and has a novel mutation, however, variants of *XK* often appear to be limited to individual families. We suspect that the absence of diagnosis of XK in individuals of African ancestry is unlikely to be due to a genetic factor, and is more likely to be due to disparities in health care access and implementation.

Sporadic *de novo* pathogenic variants in *XK* are rare events among XK disease reports; gonadal mosaicism is a plausible alternative to sporadic occurrence in patients C and D.

In general, female mutation carriers appear to be asymptomatic. A few affected women have been documented (Jung et al., 2001; Gandhi et al., 2008; Ho et al., 1996), likely due to skewed X chromosome inactivation. Family 1 contained a number of women presenting with various neurological findings, some of which were atypical for XK disease, including cerebellar atrophy on brain MRI and retinitis pigmentosa. Some of these women were obligate carriers of the causative mutation, while for others (e.g. IV-8), their genetic status was unknown. The relationship of these symptoms, and the significance of a heterozygous *XK* mutation in women remains to be elucidated. Dementia obviously can be due to a large number of causes, however it was reported in the mother of two brothers with XK disease, with neuropathology showing hippocampal TDP-43 pathology and mild Alzheimer’s-type pathology (Dulski et al., 2016).

Compulsive behaviors in general are less frequently reported in XK disease, in comparison with VPS13A disease, where they tend to be more prominent (Walterfang et al., 2011; Walker et al., 2006). Various other features of both diseases can be present in both but tend to be less prominent and more variable in appearance in XK than in VPS13A disease, such as feeding dystonia (Gantenbein et al., 2011), chorea, and cognitive impairment. Conversely, features which tend to be more prominent and debilitating in XK disease than VPS13A include myopathy (Hewer et al., 2007), and cardiac disease (Oechslin et al., 2009).

With the increased availability of WGS/WES more patients are being diagnosed incidentally prior to the appearance of clinically significant neurological symptoms, however, it is of note that all of the cases reported here underwent targeted genetic sequencing, in most cases following identification of the McLeod RBC phenotype. The diagnosis of XK disease may eventually be corroborated by the combination of variable central nervous system, neuromuscular and cardiac manifestation in combination with elevated CK, although these may be absent or very mild, even in later years (Walker et al., 2007). As appropriate interpretation of these genetic results requires clinical context, it is critical that practitioners outside the realm of neurology become aware of the spectrum of symptoms potentially attributable to XK disease (for example, the minimal impairment of case VI-5 from Family 1). In particular, sleep-related symptoms are important contributing factors to morbidity and mortality and are potentially manageable. Regular cardiac monitoring should be performed, in addition to consideration of implications for blood transfusion (Jung et al., 1993). It appears that all mutations so far identified as pathogenic will eventually lead to clinical manifestations, albeit variable in severity (Walker et al., 2007) (i.e. disease penetrance is complete and only mitigated by patient age). Identification of mutation carriers at a young age drives the incentive to develop molecular therapies prior to clinical disease manifestation, to ensure safety of blood transfusions is needed, and also to obviate unnecessary investigations such as liver or muscle biopsy.

## Data Availability

The original contributions presented in the study are included in the article/supplementary material, further inquiries can be directed to the corresponding author.
